# High electrochemical and mechanical performance of zinc conducting-based gel polymer electrolytes

**DOI:** 10.1038/s41598-021-92671-5

**Published:** 2021-06-24

**Authors:** Isala Dueramae, Manunya Okhawilai, Pornnapa Kasemsiri, Hiroshi Uyama

**Affiliations:** 1 Metallurgy and Materials Science Research Institute, Chulalongkorn University, Bangkok, 10330 Thailand; 2 Center of Excellence in Responsive Wearable Materials, Chulalongkorn University, Bangkok, 10330 Thailand; 3 Sustainable Infrastructure Research and Development Center and Department of Chemical Engineering, Faculty of Engineering, Khon Kaen University, Khon Kaen, 40002 Thailand; 4 Department of Applied Chemistry, Graduate School of Engineering, Osaka University, Suita, Osaka 565-0871 Japan

**Keywords:** Batteries, Batteries

## Abstract

Zinc ionic conducting-based gel polymer electrolytes (GPEs) were fabricated from carboxymethyl cellulose (CMC) and three different zinc salts in a mass ratio ranging within 0–30 wt%. The effects of zinc salt and loading level on the structure, thermal, mechanical, mechanical stability, and morphological properties, as well as electrochemical properties of the GPEs films, were symmetrically investigated. The mechanical properties and mechanical stability of CMC were improved with the addition of zinc acetate, zinc sulphate, and zinc triflate, approaching the minimum requirement of a solid state membrane for battery. The maximum ionic conductivity of 2.10 mS cm^−1^ was achieved with the addition of 15 wt% zinc acetate (ZnA), GPE_A_15. The supported parameters, indicating the presence of the amorphous region that likely supported Zn^2+^ movement in the CMC chains, were clearly revealed with the increase in the number of mobile Zn^2+^ carriers in FT-IR spectra and the magnitude of ionic transference number, the decrease of the enthalpy of fusion in DSC thermogram, and the shifting to lower intensity of 2*θ* in XRD pattern. The developed CMC/ZnA complex-based GPEs are very promising for their high ionic conductivity as well as good mechanical properties and the ability for long-term utilization in a zinc ion battery.

## Introduction

The growing demand of sustainable energy storage with the environmental concerns has stimulatingly developed for various advanced energy storage technologies^1^. Although a striking source of lithium-ion batteries (LIBs) provides high energy density and rechargeable properties for various applications, the disadvantages including high cost, complication of lithium electrodes management, and safety restrictions have forced researchers to look for alternatives, such as sodium-, magnesium-, and zinc-based rechargeable batteries^2^. Rechargeable zinc-based batteries (RZBs) are compelling alternative batteries of the renewable energy sources due to plentiful resources, cost effectiveness, less toxicity, being inflammable, promising energy density, and being environmental friendlier^1^. In order to develop high-performance RZBs with efficient ionic conductivity and separate the electrodes for avoiding short circuiting, one essential key is the seeking of a suitable electrolyte system, which governs the battery electrochemistry. Liquid electrolytes (LEs) such as aqueous neutral or mildly acid and alkaline solutions (KOH or NaOH) have been generally utilized in the RZBs^1^. However, the negative impacts of dendrite growth and corrosion could unavoidably take place for long-term cycling process. Recently, the replacement with solid polymer electrolytes (SPEs) has been reported for both LIBs and RZBs with different outstanding properties^3–7^.

Gel polymer electrolytes (GPEs) are regarded as proficient replacement candidates, which carry the valuation of SPEs and electrochemistry effectiveness of LEs. Furthermore, they exhibit several benefits, such as various accessibility shapes, increase of charge–discharge rate, light weight, low cost, and high power density^8–10^. Recently, several GPEs in a combination with various zinc salts have been reported^11–13^. A biodegradable polymer matrix of poly-ε-caprolactone (PCL) has been utilized as a host polymer for zinc triflate (Zn(Tf)_2_). The maximum ionic conductivity of 1.1 × 10^–4^ S cm^−1^ was achieved through incorporating an 1-ethyl-3-methylimidazolium bis(trifluoromethylsulfonyl) imide ionic liquid ((EMIM)(Tf_2_N) based IL), causing loss of the mechanical properties and lack of compatibility with the electrodes^11^, hence affecting the performance and safety of the battery. Moreover, the “syneresis effect” might happen in the GPEs, which is the phenomenon of solvent leakage consequent to long storage^14^. As in the literatures, zinc sulphate (ZnSO_4_) and Zn(Tf)_2_-based solutions offered favourable environments for the battery system. In particular, the Zn(Tf)_2_ electrolyte exhibited high stability^15^, supposedly due to a reduced solvation effect because of the presence of bulky CF_3_SO_3_^−^ anions. However, the decrease in the ionic conductivity is commonly encountered at high Zn(Tf)_2_ concentration. Furthermore, Zn(Tf)_2_ is of a much higher cost than the others, which may be an obstacle in large scale process^16^. Therefore, different anions of zinc salts have been comparatively considered with Zn(Tf)_2_ in this research, for good environmental compatibility of zinc acetate (ZnA; ZnC_4_H_6_O_4_)^17^ and low cost with high solubility in water of ZnSO_4_^18^.

Carboxymethyl cellulose (CMC) is a cellulose derivative consisting of *β*-linked glucopyranose residues, dangling the partial hydroxyl groups substituted with carboxymethyl (–CH_2_COO–) groups. A polyelectrolyte complex can form via a strong linkage between this anion and opposite charged constitutes^19^. Moreover, CMC shows a good potential host polymer for conducting path due to the swelling ability via H-bonding with multiple carboxyl groups, which can form hydrogels by itself without any crosslink agents. Furthermore, the characteristic of semi-crystalline material exhibits an excellent film forming ability^20^. In addition, CMC is a biodegradable polymer, with low production cost and low environmental toxicity.

Therefore, the development of GPEs has been deeply researched in the present work to achieve high ionic conductivity with excess standard of mechanical properties for the battery as well as the safety for long-term usage. The GPEs were formed through incorporating different anions of mildly zinc salts in CMC without adding any plasticizers or IL. The structure, mechanical, thermal, mechanical stability, and electrochemical properties were studied as a function of dopant salts concentrations.

## Materials and characterization methods

### Materials

Sodium CMC was purchased from the Changshu Wealthy Science and Technology Co., Ltd., China (white powder, viscosity of 2580 mPa × s for 2 wt% solution at 25 °C, degree of substitution = 0.76). Zinc triflate Zn(Tf)_2_, zinc acetate (ZnA), and zinc sulphate (ZnSO_4_) were purchased from Sigma-Aldrich Corporation. The deionized water was used as a solvent for the whole fabrications and operation process.

### Formation of the CMC/zinc salt complex-based GPEs

The GPEs were fabricated by a facile solution casting. The different zinc salts were dissolved in 1 wt% CMC aqueous solution with a weight fraction of 0–30 wt%. The solutions were cast into acrylic blocks and dried at 50 °C, approaching the constant weight. The obtained GPEs are designed as GPE_A_x, GPE_S_x, and GPE_T_x for the GPEs, using ZnA, ZnSO_4_, and Zn(Tf)_2_, respectively (where x is the concentration of zinc salts). The quality of film depends on salts and salt contents. However, we control the area and thickness for each experimental procedure.

## Characterization methods

### Characterization of the chemical structure of the materials

The chemical structures of the CMC and GPEs were investigated using Fourier-transform infrared spectroscopy (FT-IR). A model of Horiba FT-IR 720 spectrometer equipped with an attenuated total reflectance accessory was utilized. All samples were scanned at a resolution of 4 cm^−1^ within the spectral range of 4000–650 cm^−1^. The obtained results were subtracted with the background spectra.

### Testing of the mechanical properties

The mechanical properties of GPEs were characterized at room temperature with a universal testing machine (Introns Co., Ltd., model 5567). The GPE samples with a dimension of 8 mm × 80 mm × 4 mm (length × width × depth) were prepared. A tensile mode was selected with a supporting span of 4 mm and a crosshead speed of 4 mm min^−1^.

### Investigation of the rheological properties

Viscoelastic behaviour of swollen samples was performed with a TA instrument METTLER STARe model. The GPE samples were tested through the use of a parallel plate with a diameter of 25 mm. A linear viscoelasticity region was initially confirmed via the dynamic strain sweep testing. The shear modulus (G′) and loss modulus (G″) as a function of frequency were investigated, using a strain of 0.01% and normal controlled force of 1.0 N at room temperature.

### Measurement of the thermal properties

Melting temperature and enthalpy of fusion of samples were examined through the use of differential scanning calorimetry (DSC; METTLER STARe model). The aluminium pan was utilized as the container for the characterized samples. The measurement was carried out in temperatures ranging from 30 to 300 °C at a heating rate of 10 °C under a N_2_ flow of 50 mL min^−1^, using an empty pan as the reference.

### Measurement of the thermo-mechanical properties

The temperature dependence on the mechanical stability and glass transition temperature of CMC and the optimized samples of each GPEs series was studied via TA Instruments (DMA; METTLER STARe model) with the tensile mode. A temperature sweep from 30 to 250 °C was conducted with a heating rate of 2 °C min^−1^ and frequency of 1 Hz. The initial strain and preload force were 0.01% and 0.1 N, respectively.

### X-ray diffraction (XRD) measurements

The crystallinity of samples was investigated by a wide-angle X-ray diffractometer (model PW3710, Philips, The Netherlands). The Brukr D8 Advance diffractometer equipped with monochromatic Cu K_α_ radiation (λ = 1.542 A) at 40 kV and 30 mA was utilized with a step size of 0.2° from 5° to 80° at room temperature.

### Investigation of the material morphology

The surface structures of CMC and the optimized samples of each GPEs series were observed using scanning electron microscopy (SEM) (Hitachi SU-4800). The field emission scanning electron microscope was accelerated at a voltage of 3.0 kV and emission current of 10 mA. The fractured surfaces of the samples were sputter-coated with gold before measurement.

### Electrical impedance spectroscopy (EIS)

A potentiostat/galvanostat (PSTrace4 Palm Sens) was utilized for investigating the electrochemical properties. The measurements were performed on an applied 10 mV AC potential from 100 kHz to 10 mHz under the open circuit potential. The Zn/GPEs/Zn cell was constructed by inserting the thin GPEs films with/without the separators between blocking electrodes made of stainless steel. The film thickness and active area were approximately 100–120 μm and 2.0114 cm^2^, respectively. Transference number measurement was performed using dc polarization method, using chronoamperometry. A polarization voltage of 10 mV was applied across the sample and the initial maximum current *I*_0_ and steady state current *I*_s_ were recorded.

### Measurement of charge–discharge cycles

The voltage response on the electrochemical compatibility of CMC and GPE_A_15 films was investigated and recorded as a function of time at room temperature for long-term zinc charge/discharge cycles, through the use of a Neware testing system (Shenzhen Neware CT-4008). The charge–discharge cycles of the symmetric Zn/GPEs/Zn cells were carried out at a current density of 0.5, 2.0, 4.0, 6.0, 8.0, and 10.0 mA cm^−2^ for 150 h. Then, the current density was tuned to the initial value of 0.5 mA cm^−2^ for 75 h.

## Results and discussion

### Chemical structure of the CMC/zinc salts complex

**Figure 1:**
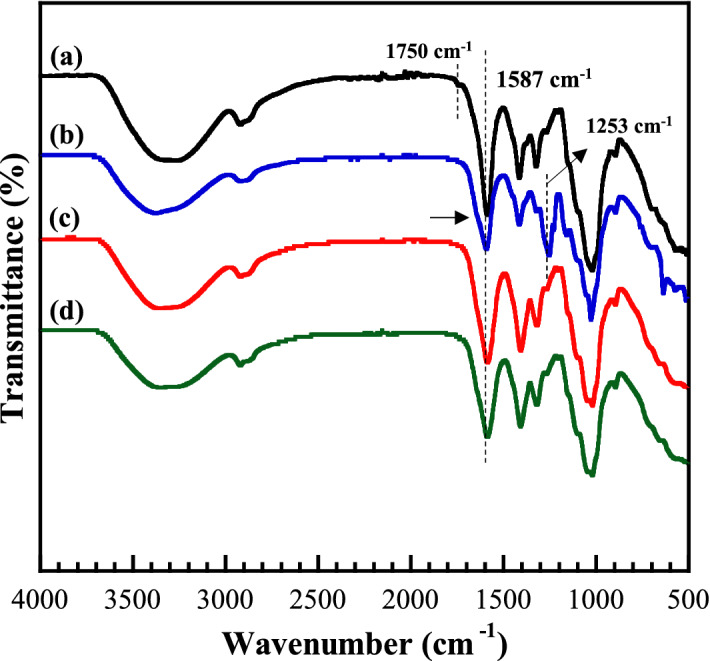
Representative FT-IR spectra of CMC/zinc salt complex: (**a**) pure CMC, (**b**) GPE_A_15, (**c**) GPE_S_15, and (**d**) GPE_T_15.

FT-IR spectroscopy was utilized for understanding the alterations of CMC functional groups when the zinc salts were introduced. The FT-IR spectra of GPEs with 15 wt% zinc salts are represented, in comparison with CMC as shown in Fig. 1. Similar to our previous study^5^, the functional groups of the host polymer showed a broad band of O–H stretching vibration at around 3296 cm^−1^ and C–H stretching vibration at approximately 2920 cm^−1^, as well as a small peak of the C=O stretching vibration at approximately 1750 cm^−1^ and the asymmetric stretching vibration of the carboxylate groups at 1587 cm^−1^. Both –CH_2_ scissoring and COO− symmetric stretching vibrations were presented at 1415 cm^−1^. The peaks of 1324 cm^−1^ and 1023 cm^−1^ were attributed to the O–H bending vibration and the ether groups, respectively^5,21^. In the literature, transmittance bands of zinc acetate salts were marked at 1056 cm^−1^, 1078 cm^−1^, 1635 cm^−1^, 1731 cm^−1^, and 620 cm^−1^, corresponding to C-CH_3_, CH_3_ bending vibration, deformation vibration of Zn–O, C=O, and the acetate anion twisting and scissoring, respectively^22^. Four fundamental vibrations of a free sulphate ion, namely, a non-degenerate mode (υ1), a doubly degenerate mode (υ2), and triply degenerate vibrations (υ3 and υ4), have been generally reported at 981, 613, and 1104 cm^−1^, respectively^23^. The characteristic of the free triflate anion is noticed at 1231 and 1195 cm^−1^ for symmetric and asymmetric CF_3_^−^ stretching vibrational mode, 640 cm^−1^ for asymmetric SO_3_ bending group, and 1032 cm^−1^ for symmetric SO_3_^2−^ stretching vibrational mode^24^.

The functional groups of pure CMC and ions of salts still existed in the GPEs system as demonstrated in Fig. 1b–d. The band corresponding to the hydroxyl group at the center of 3296 cm^−1^ in the FT-IR spectrum of CMC becomes boarder and splits to the overlapped peak in the GPEs, indicating the involving of –OH groups in the complexation. The characteristic peak at 1750 cm^−1^ of C=O stretching of the ester group in aromatic ring of CMC disappeared when the complex of CMC and zinc salts was formed, whereas the FT-IR peak at 1587 cm^−1^ corresponding to the carboxyl group (COO^−^) of CMC slightly shifted with the decrease and broadening of intensity for GPEs. In addition, a small overlapped new shoulder peak was clearly observed as pointed with the arrows. The change of this probable site for interaction with zinc salts probably contributed to the lone pair of electrons at oxygen atom, donating to Zn^2^^+^ ions for the coordination to form the C=O ^…^ Zn^2+^ bond^25^. The overlapping peaks at the ranges from 1200 to 950 cm^−1^ were observed, corresponding to the combination of free ions and contact ion pairs/higher aggregates. Furthermore, the free ions are most expected for the complexation. However, the formation of ion pairs/higher aggregates is inevitable, even at low concentrations of the salt^12^. Interestingly, the appearance of new peaks at the center of 1253 cm^−1^ is presented in the GPE_A_15 as seen in Fig. 1b, corresponding to the C–O group of anions in the zinc acetate, which could refer to the appearance of free Zn^2+^ conducting charges and participate in the enhancement of the conduction process.

### Mechanical properties of the CMC/zinc salts complex

**Figure 2:**
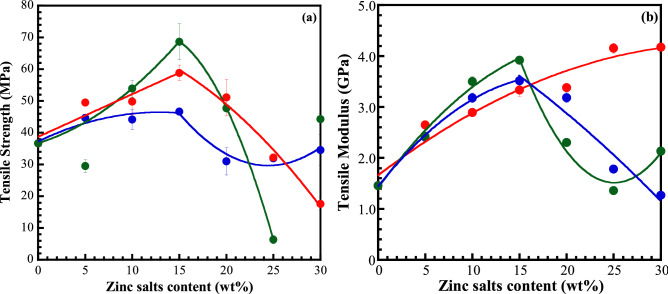
Tensile strength (**a**) and tensile modulus (**b**) of CMC with different zinc salts and concentration. (Blue) GPE_A_x system, (red) GPE_S_x system, and (green) GPE_T_x system. Data derived from 5 independent repeats. The curves are drawn as guides to the eyes.

The mechanical properties of a polymer electrolyte are also critical, intrinsic factors that affect the safety and large-scale manufacture of batteries. The tensile strength and modulus of the CMC and GPEs films are displayed in Fig. 2. The tensile strength and tensile modulus were found to be 35.6 MPa and 1.4 GPa, respectively, for CMC film. Similar behaviour was observed in tensile strength and modulus for the almost GPEs system. Both properties significantly increased with increase of zinc salt content and reached the maximum value at 15 wt% zinc salt loading. They went drastically on decreasing after that for GPE_S_x and GPE_T_x system. The tensile strength of GPE_A_x system slightly increased with the maximum value at 15 wt% ZnA and suddenly drop at 20 wt% with a plateau at higher ZnA content. Whereas the tensile modulus gradually increases with an increase of ZnSO_4_ content. Excepting, the tensile strength of GPE_T_5 and GPE_T_30 is out from anticipation, suggesting the fluctuation of five independent repeats for the calculation values.

At the appropriated zinc salt content of 15 wt%, the tensile strength was found to be 46.7, 58.9 and 68 MPa for the GPEA15, GPE_S_15 and GPE_T_15, respectively. The tensile modulus of GPEs outstandingly rose 2.29–2.42 folds in comparison with the CMC host polymer. This difference is probably attributed to the difference in shape and physical properties of each zinc salts. From the results of optimum point, the GPE_A_x system showed slightly lower tensile strength and modulus than the other, indicating more flexibility of molecular chains. It is beneficial characteristic to ionic conductivity. At the lower zinc salt loading, the enhancement of the mechanical properties of the GPEs films can ascribe to the effective reinforcing impact of the fair distribution of zinc salts in the CMC, which efficiently restricts the mobility of the CMC chain during deformation. This might be also attributed to the formation of an intermolecular bonding between both containing cations and anions constituent materials. They could participate in the carboxyl and hydroxyl groups of CMC in order to form the strong intermolecular bonding and electrostatic interactions. The GPEs materials become stiffer than CMC film. Contrastingly, when the CMC was replaced with the excessive zinc salt content, they were not properly dispersed in the CMC. This led to weakness of the salt/CMC interaction and the reduction of stress transfer efficiency due to the formation of the agglomerates. Consequently, the GPEs showed poor mechanical properties, resulting in the brittle of materials. However, the incorporation of zinc salts into the CMC matrix can greatly enhance the mechanical properties of the films, which have rarely been reported in the other SPEs and GPEs systems^26^. Moreover, the suitable electrolytes of GPEs for cell assembling approach the minimum requirement of a solid state membrane for battery (should be ≥ 30 MPa for tensile strength)^27^, except for the GPE_S_30 and GPE_T_25. Therefore, the results confirmed that the GPEs can resist the stress during the cell assembly and protect the dendrite growth during the charge/discharge process.

### Mechanical stabilities of the swollen CMC/zinc salts complex

**Figure 3:**
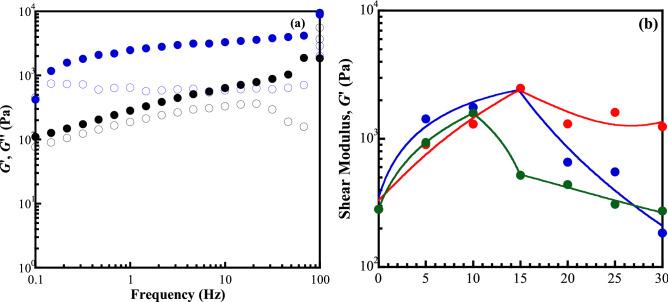
Rheology characterization (**a**): (solid symbols) shear modulus, *G′*, and (hollow symbols) loss modulus, *G″* of CMC host polymer in black colour and GPE_A_15 sample in blue colour. The shear modulus at 1 Hz (**b**) of (blue) GPE_A_x, (red) GPE_S_x, and (green) GPE_T_x systems. The curves in (**b**) are drawn as guides to the eyes.

GPEs contain some moisture, rendering poor mechanical property due to the possibility of GPEs shrinkage. This is a great obstacle to their application. In order to prevent the short circuit of cells in the long-term utilization, the deformation of CMC and GPEs was observed in the swollen state with the rheological test. The percentage of water content in the materials was equally controlled. The shear modulus (*G′*) and loss modulus (*G*″) as a function of frequency for the CMC and GPE_A_15 at room temperature are revealed in Fig. 3a. The *G′* and *G″* changed as the frequency increased. The CMC and GPE_A_15 became more solid at the higher applied frequency and otherwise had liquid-like behaviour at lower frequencies. The *G′* was much higher than the *G″* over the whole frequency range, indicating that the materials had a stable structure with the elastic characteristic under the swollen state. The *G′* and *G″* of GPE_A_x showed a higher magnitude than those of the CMC sample for the whole measured frequency. In the case of CMC sample, there was a larger slope of *G*′ and smaller difference in G′ and G″ magnitudes than GPE_A_15 sample. It could be interpreted that the molecular structure of CMC becomes more sensitive to the shear forces. Therefore, the CMC does not allow longer polymer chains to rearrange in the given time scale and can be deformed by thermal disruption. Otherwise, the GPE_A_15 exhibited more stable structure with the response to environmental changes. Interestingly, the *G″* of GPE_A_15 was almost constant with the changes of frequency as stronger interactions were expected in the molecular chains. Since the *G′* increased with frequency, the *G′* of all samples was compared at low frequency of 1 rad/s as shown in Fig. 3b. The *G′* of GPEs system demonstrated the concentration dependence with the same tendency with mechanical properties.

### Thermal properties of the the CMC/zinc salts complex

**Figure 4:**
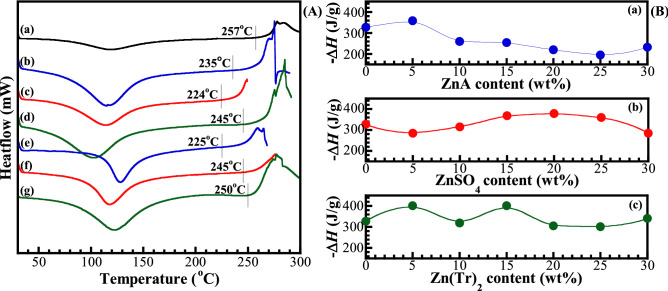
Representative DSC thermograms (**A**) of samples: (**a**) CMC, (**b**) GPE_A_5, (**c**) GPE_S_5, (**d**) GPE_T_5, (**e**) GPE_A_25, (**f**) GPE_S_25 and (**g**) GPE_T_25. Enthalpy of fusion of samples (**B**) in different zinc salts and concentration: (**a**) GPE_A_x, (**b**) GPE_S_x, and (**c**) GPE_T_x systems.

DSC analysis was carried out to determine the first and second order thermal transitions like melting (*T*_m_), crystallization (*T*_c_), and glass transition temperature (*T*_g_) phenomena, as well as to evaluate the possible changes in the film crystallinity. The DSC thermograms of the neat CMC and 5 wt% and 25 wt% zinc salt contained-GPEs films were represented in Fig. 4a. As the thermal scanning, two distinct peak regions were clearly observed, the complex endothermic and exothermic behaviours. The first endothermic peak is attributed to a melting transition for crystalline fraction, and also the dehydration of absorbed water in CMC film. Although the CMC film was dried before characterization and purged with nitrogen gas before thermal scanning. It still contained amounts of water molecules due to the strong hydrophilicity. The peaks corresponding to the *T*_m_ slightly shift from 115 °C of CMC as clearly seen in Figure S1. For this thermal transition of broad endotherm peak, the *T*_g_ has been reported as 78.21 °C^28^, 77.39 °C^29^ and 81 °C^30^. Whereas, Shahbazi, et al. have reported the *T*_g_ of CMC around 143.2 and 190.5 °C, which were detected after the endothermic transition^31^. The exothermic peak was incompletely performed due to the combustion of materials, which corresponds to a transition due to crystallization. The onset temperature of this transition was found to be 257 °C for CMC and shifted to the lower value of 224–250 °C for CMC containing zinc salts. This could be suggested the crystalline of CMC is reduced with the addition of salts.

The crystallinity of the materials is determined in this transition. However, the exothermic peak reflects the initiation of thermal destruction, which cause the major degradation, depolymerization of pyrolytic decomposition of CMC and polysaccharide backbone^32^. Since the ionic conductivity is related to the overall change in the crystalline. Therefore, we would alternatively observe at the endothermic peak from the area under the DSC thermograms curves, which is related to the change of the enthalpies of crystalline fraction.

Pure CMC is relatively assumed to be 100% crystalline, and the relative percentage of crystallinity (*X*_c_) was calculated based on the following equation.

1 $$ X_{{\text{c}}}  = \left( {\frac{{\Delta H_{{\text{m}}} }}{{\Delta H_{{\text{m}}}^{0} }}} \right) \times 100\%  $$

where $\Delta{{H}}_{\text{m}}^{{0}}$ is the standard enthalpy of fusion of pure CMC (i.e., 327 J g^−1^) and $\Delta{t{H}}_{\text{m}}$ is the enthalpy of fusion of the GPEs. In Fig. 4b, $\Delta{t{H}}_{\text{m}}$ decreases with the addition of zinc acetate, otherwise for GPE_S_x and GPE_T_x systems. *X*_c_ was found to be 59.8–79.3% for GPE_A_x system. The decrease of *X*_c_ contributes to the increase of the amorphous phase. The crystalline region of CMC can be inhibited by ZnA, which interrupts the alignment of the polymer chains and decreases the crystallinity of the system^33^. The results are in agreement with the decrease of the onset temperature at the exothermic peaks. However, when the ZnSO_4_ and Zn(Tf)_2_ were added, *X*_c_ increases. This is possibly due to the particle aggregation of ZnSO_4_ and Zn(Tf)_2_, which leads to increase in crystallinity^34^.

### Thermo-mechanical properties of the CMC/zinc salts complex

**Figure 5:**
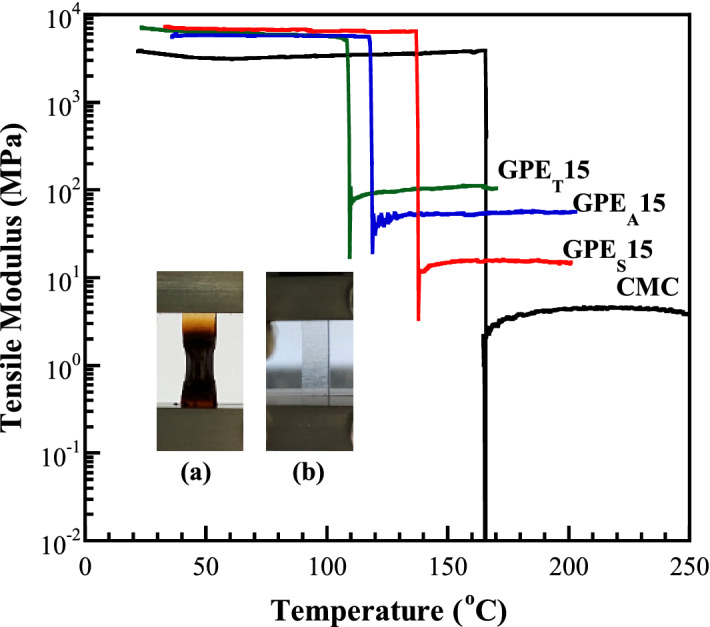
Tensile modulus as a function of temperature of CMC and GPEs composed of 15 wt% different zinc salts; inset: the physical appearance of (**a**) CMC and (**b**) GPEs.

Dynamic mechanical analysis (DMA) has been utilized for investigating the temperature dependence on mechanical stability. Moreover, among the influencing factors on the ionic conductivity of GPEs, *T*_g_ is also detected. Figure 5 shows the tensile modulus (*E′*) variations of pure CMC and GPEs as a function of temperature. The *E′* showed the plateau as the increase of temperature, which the GPEs exhibited higher than that of CMC. Consequently, the *E′* drastically changed from their glassy to rubbery nature which could be presented as a *T*_g_. In this state, the amorphous region starts to develop large-scale coordinated motion with the continual heating. The free volume increases, leading to the initiation of localized bond and side chain movements. The *T*_g_ of CMC was observed at 165 °C, which corresponds with the DSC result in the previous study^31^. The *T*_g_ decreased to be 115, 137 and 106 °C for GPE_A_15, GPE_S_15 and GPE_T_15, respectively, implying the increase of amorphous phase which facilitates the improvement of ionic conductivity. On the other transition, CMC decomposed after this step change as shown in the physical appearance in the inset of Fig. 5, which related to the incomplete exothermic peak in the DSC results.

With the results of FT-IR, the existence of the coordinated interaction between ether oxygen and zinc cations supports the increase of *T*_g_. However, the *T*_g_ value decreases, resulting in the weakening of the coordinated interaction by the formation of the ion pairs and/or higher-order ionic aggregates. Moreover, the larger anions could act as the plasticizer, which increases in chain mobility, yielding the decrease in *T*_g_ of polymer-salt complexes^35^.

### Phase structure, microstructure arrangement, and morphology of the CMC/zinc salts complex

**Figure 6:**
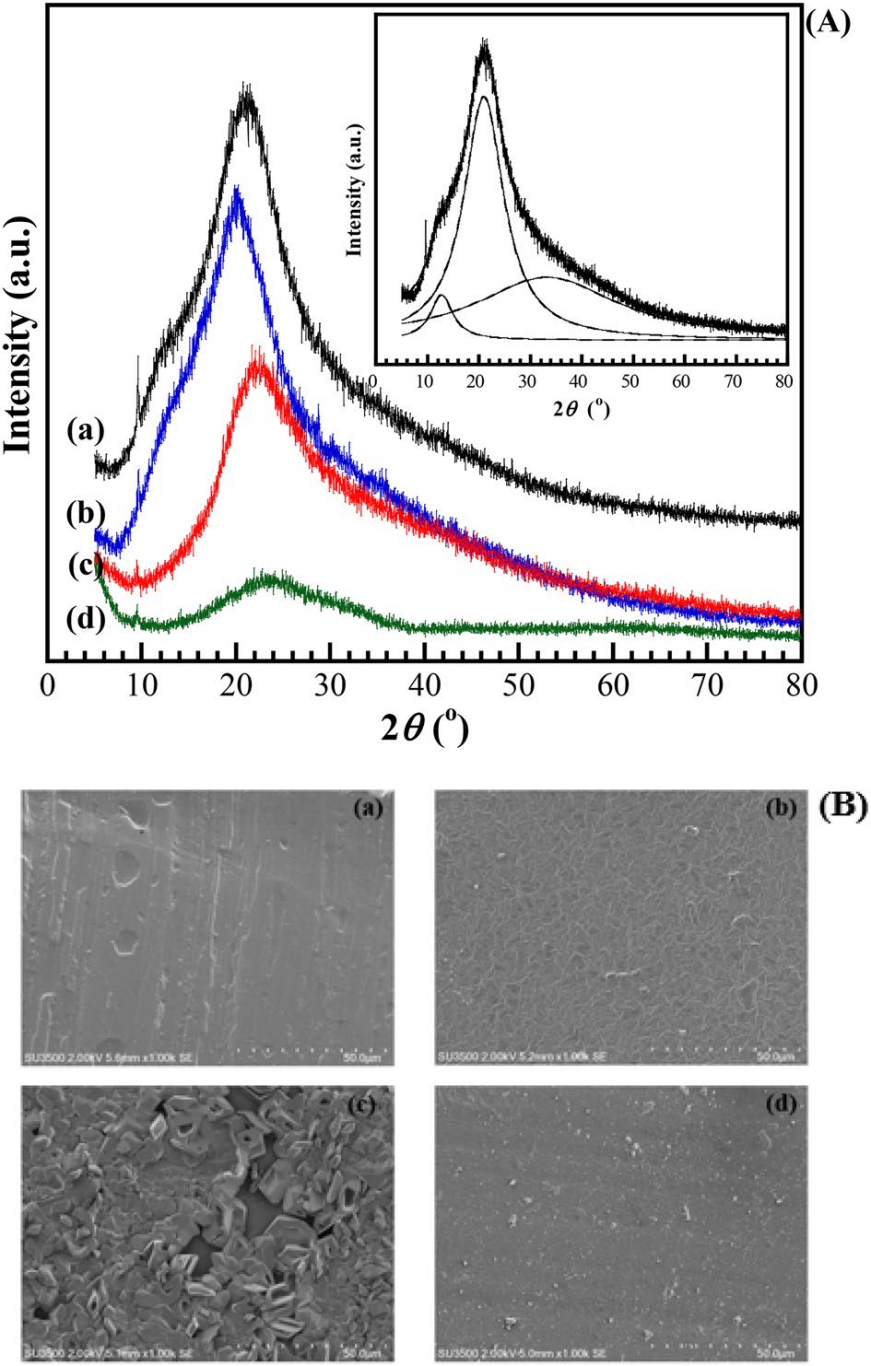
Representative XRD patterns (**A**) of (**a**) CMC, (**b**) GPE_A_15, (**c**) GPE_S_15, and (**d**) GPE_T_15; inset: deconvolution of CMC peak. Representative SEM micrographs (**B**) (×1000 magnification) of (**a**) CMC, (**b**) GPE_A_15, (**c**) GPE_S_15, and (**d**) GPE_T_15.

X-ray diffraction (XRD) characterization was conducted in order to examine the impact of different dopant salts on the phase structure and microstructure arrangement of CMC host polymer. XRD spectra of pure CMC and GPEs with different zinc salts are shown in Fig. 6A. The X-ray diffraction pattern of CMC host polymer exhibits a clear broad peak at 2θ = 20.9° and two overlapped broad shoulders as clearly seen in the inset of Fig. 6A, demonstrating the semi-crystalline characteristic of CMC polymer. The obtained results of GPEs have more amorphous morphology in comparison with the CMC host polymer, as evidenced by the lower intensity and wider diffraction peaks. It is worth noting that the XRD intensity peak of GPE_A_15 shifts to lower diffraction, whereas those of GPE_S_15 and GPE_T_15 shift to higher diffraction of CMC. Moreover, the main and overlapped intensity peak of the first case equally remained as CMC, and it became two peaks and single peak for GPE_S_15 and GPE_T_15, respectively. The sharp peaks of highly crystalline character of zinc salts completely disappear in almost all the GPEs complexes: the 2*θ*  = 11–12° for ZnA^36^, the 2*θ*  = 17.4° for Zn(Tr)_2_^37^, and multi-sharp peaks for ZnSO_4_^38^, thereby indicating the feasibility of an absolute complexation and complete dissolution of zinc salts in the GPEs. The disappearance of crystalline peaks led to the reduction in the energy barrier to the segmental motion of the polymer electrolyte, resulting in the greater diffusion of ions and inducing the highest ionic conductivity as well^39^.

Based on the position of the main peak in the XRD patterns, the distance between polymer chains in the amorphous phase can be analysed through identifying the average interchain spacing (< *R* >) following Eq. (2)^40^:

2 $$\langle{R}\rangle {=}\frac{{5}}{{8}}\left(\frac{{ \lambda  }}{{sin}{ \theta  }}\right)$$

where *λ *is the wavelength of radiation, *θ* is half of the scattering angle measured from the incident beam, and the 1/sin*θ* term in Bragg’s law acts as an amplification factor. The < *R* > was found to be 5.31, 5.52, 4.94, and 4.59 for the host polymer, GPE_A_15, GPE_S_15, and GPE_T_15, respectively. This means that their packaging in the amorphous phase shows some differences. The lowest value of interchain spacing indicates its more compact structure^41^. On the other hand, the highest < *R* > of GPE_A_15 implied its less compact structure or more free volume, which increased the mobility (*μ*_*i*_) as well as the numbers (*n*_*i*_) of charge carriers^36^, assisting in the ion conduction process. The XRD data support the enhancement in ionic conductivity of the samples, which agrees with the present conductivity measurements as well.

The scanning electron microscopy (SEM) is one of the most versatile instrumental tools for evaluating and examining the microstructure morphology of conducting surfaces. The surface morphology of the CMC and GPEs with 15 wt% of zinc salts films was observed through the use of SEM. Figure 6B presents the morphology of the CMC with different zinc salts. The pure CMC displayed a combination of a coarse and smooth surface as revealed in Fig. 6B-a. The smooth surface could be attributed to the amorphous region in the polymer structure that is suitable to act as a host polymer in the further expansion of the GPEs^5^. The surface morphology then displayed a uniform dispersion of salts in polymer with the exhibition of somewhat different morphologies for GPE_A_15 and GPE_T_15 as presented in Fig. 6B-b,B-d, respectively. There is no obvious phase separation, which means that the CMC polymer is very compatible with the salts and has good compatibility between two components. These homogeneous dispersions tend to provide a good pathway for fast zinc ionic transport, thereby leading to an enhanced ionic conductivity. However, it seems like there is clear evidence of the non-homogenous dispersion of ZnSO_4_ in CMC as revealed in Fig. 6B-c. It might be the formation of crystalline aggregates of undissolved salt at that portion, which might cause a decrease in the number density of ions, and hence decreases of the ionic conductivity.

## Electrochemical properties of the CMC/zinc salts complex

### Ionic conductivity of the CMC/zinc salts complex

Ionic conductivity is an important factor of SPEs and GPEs for energy storage applications and refers to the migration of total free ion charges. EIS usually provides the data of the real resistance and the imaginary capacitance through the ability of a circuit to resist the flow of electrical current. The ionic conductivity (*δ*) of materials can be calculated from Eq. (3) as follows:

3 $$ \delta  = \frac{1}{{R_{i} }}\frac{d}{s} $$

where *d* is the thickness of the GPEs thin film, *S* is the area of electrodes contained within the GPEs film, and *R*_i_ is the total or ionic resistance.

**Figure 7:**
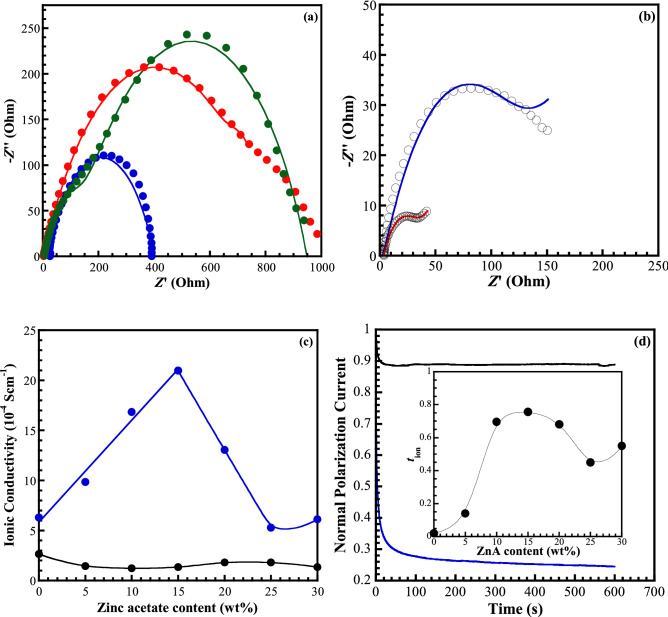
Representative Nyquist plots of the symmetrical cell with separators (**a**) of swollen CMC in (blue) ZnA, (red) ZnSO_4_, and (green) Zn(Tr)_2_ solutions and without separators (**b**) of CMC, fitting with blue line and GPE_A_15, fitting with red line. The fitting curves was performed with the equivalent circuit models using Z-view software. Ionic conductivity (**c**) with different salt content of GPE_A_x system: (black) the assembly cells with separators and (blue) without separators. Representative chronoamperometry profiles (**d**): (black) CMC and (blue) GPE_A_15. Inset: the *t*_ion_, of GPE_A_x system with different ZnA contents.

**Scheme 1:**
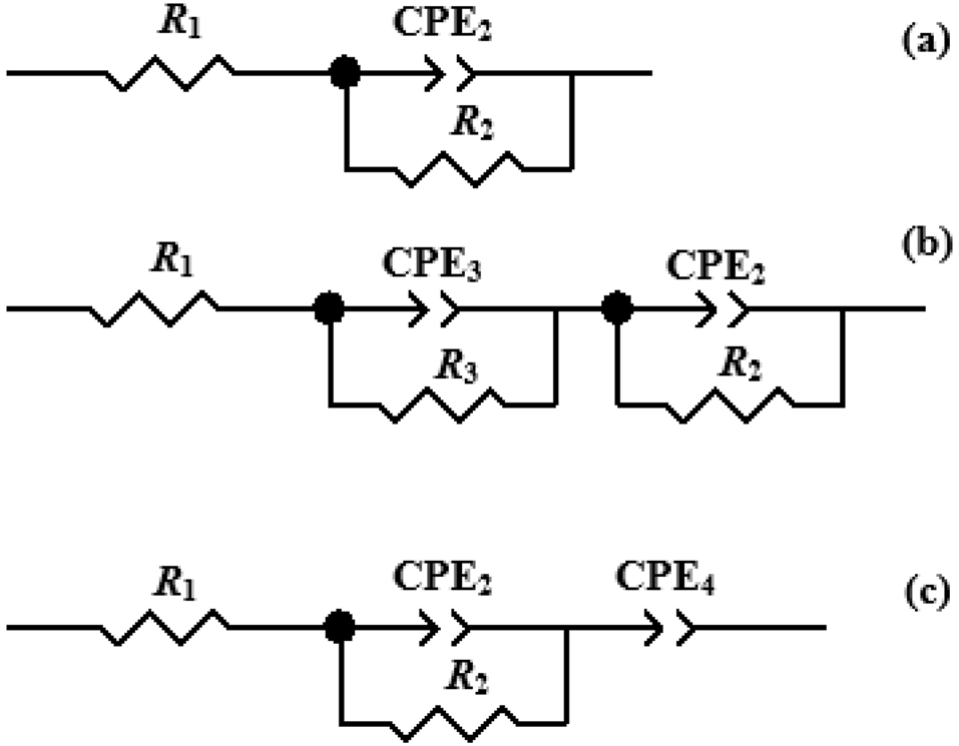
The equivalent circuit models, fitting for the assembly cells including separator of (**a**) GPE_A_x, (**b**) GPE_S_x and GPE_T_x systems and (**c**) the assembly cells without the separator of GPE_A_x system.

The ionic conductivity of free salt CMC film was reported at room temperature in the range of 6.31 × 10^–9^–1.86 × 10^–8^ S cm^−1^^42,43^, which is below the requirement of the promising candidate for ion conducting material in energy storage/conversion devices^44^. Therefore, the ionic conductivity of CMC would be improved through considering the suitable salts to form the polymer electrolyte for practical applications. First, the host polymer was swollen in the different solutions of zinc salts, ZnA, ZnSO_4_, and Zn(Tf)_2_, before assembling the symmetric cells to check the improvement of ionic conductivity, compared to free salt CMC film. The assembly cell has been subjected to AC impedance measurement. The measured real and imaginary parts are presented as the Nyquist plots in Fig. 7a,b, which are generally divided into two regions that are a semicircle at high frequency and a spike at low frequency. The differentiating characteristics of the Nyquist plots were found in this work, however, the same configuration was presented in each series. For instance, an incomplete-semicircle appeared in CMC, including the ZnA. Whereas, the incomplete-semicircles of CMC comprising the ZnSO_4_ and Zn(Tr)_2_, however, were depressed and elongated, suggesting the presence of a pair of overlapped semicircles. The spikes were hindered in the case of the assembly cell, including the separators. Therefore, the interpretation and quantification of the impedance spectra require the different appropriate equivalent electric circuit models to determine an accurate ionic conductivity as shown in the Scheme 1 using Z-view software. Considering the whole parameters, the presence of resistances, *R*_1_, which shift from the origin of real axis consists of the series resistance (*R*_s_) and bulk resistance (*R*_b_). This resistance originates from the bulk resistance (*R*_b_) of the polymer electrolyte, series resistance (*R*_s_) of the connector and internal resistance of the electrode for ion diffusion as well as ohmic loss^45^. All systems show almost similar resistance, *R*_1_ in the range of 2–8 Ω. The appearance of the semicircle is described by a parallel combination of a resistor and capacitor. Since the capacitances of the cell components are not ideal values, it is possible to obtain a more accurate model by replacing them with a constant phase element (CPE). The overlap semicircles of the EIS spectra in cases of swollen CMC with ZnSO_4_ and Zn(Tr)_2_ are associated with the formation of the interfacial layer deposited on the electrode, which is generated by the irreversible electrochemical decomposition of the electrolyte^46,47^. The product of this decomposition forms a solid layer on the surface of the electrode, affect the inability of electrolyte molecules to travel through the layer to the active material surface where they could react with zinc ions and electrons^46,47^. The maximum semicircles in the middle of the impedance spectra correlate to the charge transfer process (*R*_2_), which contributes the most to the ionic resistance (*R*_i_). For non-ideal ion diffusion characteristics, the additional element of Warburg impedance (W) should be included in the equivalent circuit model^46^, however, this part was omitted because it was difficult for the fitting and also affects the accuracy of the ionic resistance. Finally, we could extract the total resistance equivalent to the ionic resistance *R*_i_ by the combination of all specific resistances and calculated the ionic conductivity using Eq. (3). As presented in the Fig. 7a, the lowest electrolyte resistance was obtained from the swollen CMC with ZnA, which could imply the lowest charge transfer resistance of the acetate swollen CMC. It can be contributed to the highest ionic capacity in comparison with the other system. The ionic conductivity of the CMC films was found to be 2.645 × 10^–4^, 1.375 × 10^–4^, and 1.061 × 10^–4^ S cm^−1^ by using ZnA, ZnSO_4_, and Zn(Tf)_2_ as the swollen salt solutions, respectively. The improvement of ionic conductivity can be attributed to the Zn^2+^ dissociation and the transportation of the ions through the CMC structure. In addition, acetate, sulphate, and triflate anions can delocalize into two, six, and four resonances, respectively, in order to form a stable structure due to the inductive effect between the electron donors and their conjugated structure, which is a way of improving the ionic conductivity^48^. Therefore, ZnA, ZnSO_4_, and Zn(Tf)_2_ salts can be utilized as the conducting substances in order to increase the ionic conductivity of CMC.

The complex of the CMC host polymer and zinc salts was afterwards fabricated for observing the ion dissociation and hopping mechanism. The magnitude of ionic conductivity was calculated from Eq. (3), ranging between 1.229 × 10^–4^ and 1.808 × 10^–4^ cm^−1^ for the GPE_A_x system, 0.914 × 10^–4^ and 1.188 × 10^–4^ S cm^−1^ for the GPE_S_x system, and 0.434 × 10^–4^ and 0.994 × 10^–4^ S cm^−1^ for the GPE_T_x system, as demonstrated in Table 1. The high ionic conductivity of GPE_A_x system can suggest that the acetate anion can dissociate easily in comparison with the other anions due to the lower electronegativity of atom in acetate anions. Therefore, more Zn^2+^ cations can be effortlessly separated from the salt compound. The *σ* values of GPEs are lower than the swollen CMC. However, this value of conductivity is found to be greater in comparison with that of the previous reports^49,50^. Polu et al.^49^ reported that the ionic conductivity of polyethylene glycol (PEG) polymer electrolytes based on ZnA salt (30 wt%) was the order of 10^–6^ S cm^−1^ at room temperature, whereas Sai et al.^50^ reported the ionic conductivity of PVA-PEMA blended polymer electrolytes doped with 10 wt% ZnTr as 2.79 × 10^–6^ S cm^−1^. The ionic conductivity of PVdF-HEP blend/Zn(Tf)_2_ polymer electrolytes improved to 10^–3^ S cm^−1^ with the addition of EMIM TFSI additives^13^, which are absent in the present work as they cause poor mechanical property.

**Table 1:** Ionic conductivity of GPEs containing CMC and different zinc salts at room temperature.

Zinc salt concentration (wt%)	Ionic conductivity (10^–4^ S cm^−1^)		
	GPE_A_x (CMC/ZnA)	GPE_S_x (CMC/ZnSO_4_)	GPE_T_x (CMC/Zn(Tr)_2_)
0	2.645	1.375	1.061
5	1.440	0.914	0.709
10	1.229	0.934	0.834
15	1.338	1.134	0.643
20	1.808	1.034	0.772
25	1.806	1.188	0.994
30	1.352	1.030	0.434

For ion-conducting materials, the conductivity of the system depends on the host polymer (containing moisture), the ionic species concentration, the ion mobility, the ionic valences, and temperature^51^, which can generally be expressed as:

4 $$ \sigma  = \sum\limits_{i} {n_{i} } q_{i} \mu _{i} , $$

where *n*_i_ is the number of charge carriers, *q*_i_ is the charge of ions, and *μ*_i_ is their mobility.

Based on the relationship, the charge mobility is assumed to be constant, and the ionic conductivity is proportional to the natural logarithm of the free ion concentration. Therefore, the conductivity should increase with zinc salt content. To clarify the expression, the Zn/GPE_A_x/Zn cells were reassembled by the dislodgement of the separators. Figure 7b shows the Nyquist plot of the reassembled cells without the separators of CMC and GPE_A_15. The same components of the equivalent circuit model as previous cell were used in the fitting, excepting a clear observation of a spike at low frequency. The Nyquist plots could be well fitted by the equivalent circuit. However, a possible source of inaccuracy for CMC sample may arise from the different morphology of CMC in the region of contact with electrode as presented by the combination of a coarse and smooth surface in the SEM result. Figure 7c depicts the ionic conductivity as a function of ZnA concentration for both systems (with and without separators). The *δ* of the Zn/GPE_A_x/Zn cells was almost independent with the ZnA concentrations, including the separator. Meanwhile, the ionic conductivity of CMC biopolymer increases with the increase of salt concentration and then continues to increase at a decreasing rate until the conductivity reaches a maximum value of 2.10 × 10^−3^ S·cm^−1^ for the GPE_A_15 and then begins to fall. The increase of *δ* could be interpreted as the increase in the number of mobile Zn^2+^ carriers, the increase of amorphous phase, and the plasticizing effect of acetate anions^52^. The plasticizer tends to weaken the coordination bonds between Zn^2+^ cations of the salt and the negatively charged oxygen of CMC (C=O–Zn^2+^) and support the dissociation of salts to be free charge carriers, rendering the enhancement of ionic conductivity^52,53^. The decrease of ionic conductivity increment implies a decrease of mobile ions. The *n*_i_ is responsible for ionic conductivity at a lower concentration of salts, and the *μ*_i_ tends to be a key parameter at higher salt concentration. The polymer chain becomes more flexible and disordering in the molecular arrangement at high salt loading, as a result of the increase of anions plasticizer. It provides the conductivity pathways for the ions to hop a great extent, resulting in an increased conductivity value^54^. The ionic conductivity values decrease at the excess concentration of 15 wt% because of the re-association of mobile charge carriers, which is correlated to ion association, interactions of salts, ion-pair formation, and also the formation of aggregates^55^. It would naturally reduce the number of mobile charge carriers^56^, contributing to the decrease of ionic conductivity. The subsequent increase of ionic conductivity for the GPE_A_30 is probably due to the re-dissociation of ion pairs and the aggregates.

The specific capacitance of the system was calculated at a frequency of the maximum ${{Z}}^{{''}}$ from the impedance data using the following equation:

5 $$ C =  - \frac{1}{{\omega Z^{\prime\prime}}} $$

where C is the capacitance, *ω* is the angular frequency (= 1/(2π*f*), and ${{Z}}^{{''}}$ is the complex impedance. The calculated value of specific capacitance (*C*_s_) from the impedance plot was found to be a maximum value of 13.79 F g^−1^ for GPE_A_15 (see in Figure S2), correlating with the ionic conductivity results. Therefore, we can conclude that GPEs, consisting of CMC and zinc acetate, contributed to two vital roles in Zn-ion battery: (1) a separator because of its rigid structure, simultaneously avoiding the electrical contact between the anode and the cathode at the same time, and (2) the medium for the ions transportation between the anode and cathode during the cell operations.

### Transference number measurement

The ionic transference number (*t*_ion_) is one key parameter for minimization the internal resistance and the concentration polarization of metal ions during the charge/discharge cycles of the battery^57^. The magnitude of *t*_ion_ is calculated from the normal polarization current versus time using the following equation:

6 $${{t}}_{\text{ion}}{=}\frac{\left({{I}}_{\text{i}}{-}{{I}}_{\text{s}}\right)}{{{I}}_{\text{i}}}$$

where *I*_i_ and *I*_s_ represent the currents at the initial and the steady states, respectively.

As the Fig. 7d, the total current gradually decreases with time at the initial state owning to the ionic species depletion in the electrolyte. The plateau is appeared at the fully depleted situation, which the cell is polarized and current flows due to the electron migration across the electrolyte and interfaces at this steady state. The electron transference number (*t*_ele_) can be defined as the ratio of *I*_s_ and *I*_i_. The *t*_ion_, of the GPE_A_x with different ZnA contents is shown in the inset of Figure 7(d). The *t*_ion_ increased as the increase of salt contents and showed the maximum value with the addition of 15 wt% ZnA, implying the highly efficient migration of Zn^2+^ ion^58^ due to the minimization of ion paring effect and the association of cation and anion.

On the other word, it indicates that the assembly cell has a good contact between the electrolyte and the electrode that will resume the migration of zinc ions and depress the interfacial resistance, which ascends significantly the ionic conductivity as revealed in the previous section. Furthermore, it benefits the practical application of all-solid-state electrolytes.

### Electrochemical compatibility of the CMC/zinc salts complex

**Figure 8:**
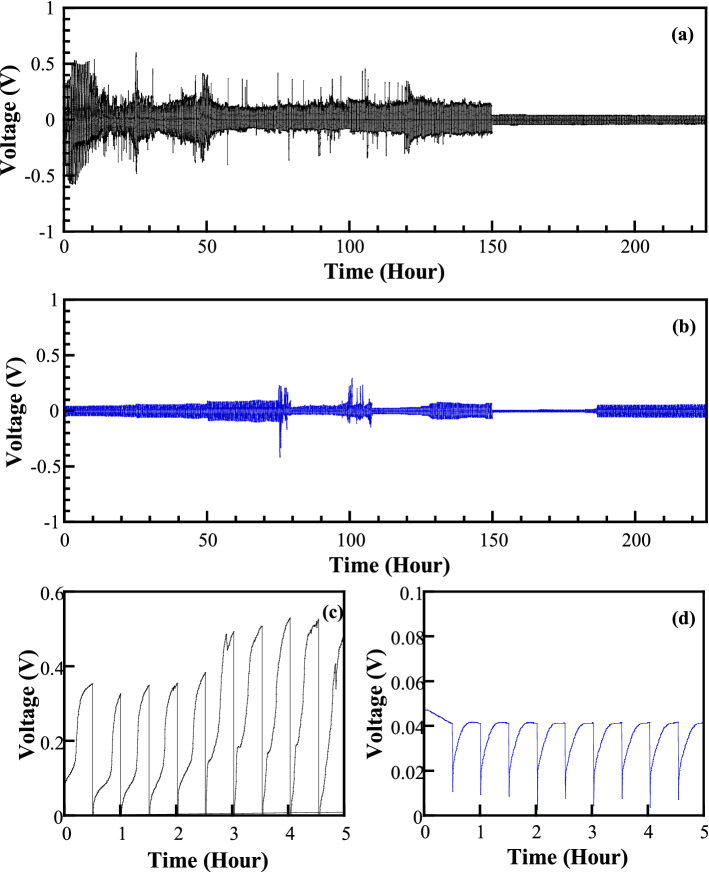
Half Zn battery cell testing for charge/discharge cycle of (**a**) CMC, (**b**) GPE_A_15, (**c**) charging process of CMC, and (**d**) charging process of GPE_A_15.

The long-term electrochemical stability was examined for preventing zinc dendrite growth of CMC-based gel electrolyte and GPE_A_15 against zinc metal. The symmetric Zn/GPEs/Zn cells were assembled and cycled at different current densities from 0.5 to 10 mA cm^−2^ and turned to 0.5 mA cm^−2^. Figure 8 displays the cycling voltage profiles and zoom-in profiles of the cells at room temperature.

The Zn stripping/plating demonstrated the fluctuation of the large voltage overpotential up to 1.20 V for a CMC-based gel polymer electrolyte (CMC-based GPE) in the current densities of 0.5–10 mA cm^−2^ as displayed in Fig. 8a. However, this recent assembling system has been developed from our previous study, where the short-circuiting of cell occurred during the cycling process after 60 h^5^. The voltage of charge/discharge suddenly drops at the last 75 h, which is probably the formation of one or more internal short circuit. Meanwhile, the symmetric Zn/GPE_A_15/Zn cell exhibits almost a stable plating/stripping process at each current density over 225 h with a small overpotential from ∼ 45 to ∼ 175 mV without any critical potential damage as presented in Fig. 8b. Moreover, the peak-to-peak voltage recovers to the similar voltage magnitude as the initial magnitudes when the current returns to 0.5 mA cm^−2^. This higher voltage overpotential reflects the higher internal resistance of the cell comprising for the symmetric Zn/CMC/Zn cell which inhibits the charge accumulation at the boundary and poor interfacial contact of CMC with the electrodes^45^. The lower polarization is observed in the GPE_A_15 electrolytes, presumably because of their higher ionic conductivity^59^. The critical short-circuit phenomenon is absent during the 225-h performance, indicating the good compatibility between the GPE_A_15 and zinc electrode. The small fluctuation of the voltage overpotential was observed for the GPE_A_15, which might be from the delocalization of two resonances in the acetate ions.

Considering the zoom-in charge process (Fig. 8c,d), the starting voltage overpotential deviates from the origin for both cells. It is ascribed to the internal resistance of cell, which is raised as a result of the disruption of the ion absorption onto the electrodes from resistance of charge transfer, bulk resistance of polymer electrolyte, and depletion of polymer electrolyte^60^. The voltage overpotential of Zn/CMC/Zn cell increases and fluctuates, indicating the greater difficulty of nucleating Zn during the plating segment, stripping Zn during the reversing segment, and the overall deposition of Zn of the cycle. Wood et al. explained this phenomenon for the Li battery that it is normally associated with the formation of thick solid electrolyte interphase (SEI) layers at the electrode and the accumulation of electronically disconnected/dead Li fibrils at the interface^47^. The curves display clearly stable voltage for the symmetric Zn/GPE_A_15/Zn cell, indicating a fast Zn^2+^ transfer through the GPE_A_15 electrolyte into the cathode side (see Fig. 8d).

The GPE_A_15 exhibits better electrochemical properties than the pure CMC-based system, which is strongly related to the higher ionic conductivity of the GPE_A_15 electrolytes. The excellent cycling performance indicates that the zinc acetate can effectively enhance the interfacial stability between GPE_A_15 electrolyte and zinc metal electrode. It is worth noting that the dopant of the zinc acetate in CMC biopolymer indeed causes lower polarization and extends the cycle life of the equipped battery. It is confirmed that the improvement of tensile strength in case of GPE_A_15 can effectively inhibit the zinc dendrite growth, preventing the internal short circuit of batteries.

## Conclusions

The development of gel polymer electrolytes (GPEs) for Zn ion battery was the aim of this work, in terms of excellent ionic conductivity as well as good mechanical properties and the ability for long-term utilization. The addition of zinc salts, that is, ZnA, ZnSO4, and Zn(Tf)2, resulted in an improvement in the mechanical properties and mechanical stability. The optimum zinc salt and polymer composition, in terms of high ionic conductivity, was found for using 15 wt% ZnA. The appearance of free Zn^2+^ conducting charges, the increase of *t*_ion_, the decrease of crystallinity, and the flexibility of polymer chain remarkably display in this composition. The ionic conductivity of GPE_A_x system increases linearly with zinc salt content, until the conductivity attains a maximum value of 2.10 × 10^−3^ S·cm^−1^ for the GPE_A_15 and then begins to fall. Furthermore, the ZnA stabilized the charge–discharge cycle performance through suppressing the dendrite formation, which otherwise causes a short circuit in the battery cell. Based on the findings, it can be concluded that the GPE_A_15, composed of 15 wt% ZnA in CMC polymer, is a promising candidate in comparison with the other GPE_A_x samples, and GPE_S_x or GPE_T_x systems, as it exhibits superb electrochemical properties.

## Supplementary Information

Supplementary Information 1.
