# Supplementary material for: High electrochemical and mechanical performance of zinc conducting-based gel polymer electrolytes
Source: Sci Rep. 2021 Jun 24;11:13268. doi: 10.1038/s41598-021-92671-5 (PMC8225769; doi:10.1038/s41598-021-92671-5)
Supplement: Supplementary file 1 — Supplementary Information 1. [file 41598_2021_92671_MOESM1_ESM.docx]

**Supplementary Information**

**High Electrochemical and Mechanical Performance of Zinc Conducting based Gel Polymer Electrolytes**

**Isala Dueramae^1^, Manunya Okhawilai^1,2,*^, Pornnapa Kasemsiri^3^, Hiroshi Uyama^4^**

*^1^Metallurgy and Materials Science Research Institute, Chulalongkorn University, Bangkok 10330, Thailand*

*^2^Center of Excellence in Responsive Wearable Materials, Chulalongkorn University, Bangkok 10330, Thailand*

*^3^Sustainable Infrastructure Research and Development Center and Department of Chemical Engineering, Faculty of Engineering, Khon Kaen University, Khon Kaen 40002, Thailand*

*^4^Department of Applied Chemistry, Graduate School of Engineering, Osaka University, Suita, Osaka 565-0871, Japan*

^*^Corresponding author email: [Manunya.o@chula.ac.th](mailto:Manunya.o@chula.ac.th)

**Figure S1.** Representative DSC thermogram of CMC sample.

**Figure S2.** (a) Frequency dependence on the imaginary part in the impedance data and (b) capacity of GPE_A_x system.
